# Towards a Science of Community Stakeholder Engagement in Biomedical HIV Prevention Trials: An Embedded Four-Country Case Study

**DOI:** 10.1371/journal.pone.0135937

**Published:** 2015-08-21

**Authors:** Peter A. Newman, Clara Rubincam, Catherine Slack, Zaynab Essack, Venkatesan Chakrapani, Deng-Min Chuang, Suchon Tepjan, Murali Shunmugam, Surachet Roungprakhon, Carmen Logie, Jennifer Koen, Graham Lindegger

**Affiliations:** 1 Factor-Inwentash Faculty of Social Work, University of Toronto, Toronto, Ontario, Canada; 2 HIV/AIDS Vaccines Ethics Group, School of Applied Human Sciences, University of KwaZulu-Natal, Pietermaritzburg, South Africa; 3 Centre for Sexuality and Health Research and Policy (C-SHaRP), Chennai, India; 4 Faculty of Science and Technology, Rajamangala University of Technology Phra Nakhon, Bangkok, Thailand; Centre for Geographic Medicine Research Coast, KENYA

## Abstract

**Objectives:**

Broad international guidelines and studies in the context of individual clinical trials highlight the centrality of community stakeholder engagement in conducting ethically rigorous HIV prevention trials. We explored and identified challenges and facilitators for community stakeholder engagement in biomedical HIV prevention trials in diverse global settings. Our aim was to assess and deepen the empirical foundation for priorities included in the GPP guidelines and to highlight challenges in implementation that may merit further attention in subsequent GPP iterations.

**Methods:**

From 2008–2012 we conducted an embedded, multiple case study centered in Thailand, India, South Africa and Canada. We conducted in-depth interviews and focus groups with respondents from different trial-related subsystems: civil society organization representatives, community advocates, service providers, clinical trialists/researchers, former trial participants, and key HIV risk populations. Interviews/focus groups were recorded, and coded using thematic content analysis. After intra-case analyses, we conducted cross-case analysis to contrast and synthesize themes and sub-themes across cases. Lastly, we applied the case study findings to explore and assess UNAIDS/AVAC GPP guidelines and the GPP Blueprint for Stakeholder Engagement.

**Results:**

Across settings, we identified three cross-cutting themes as essential to community stakeholder engagement: trial literacy, including lexicon challenges and misconceptions that imperil sound communication; mistrust due to historical exploitation; and participatory processes: engaging early; considering the breadth of “community”; and, developing appropriate stakeholder roles. Site-specific challenges arose in resource-limited settings and settings where trials were halted.

**Conclusions:**

This multiple case study revealed common themes underlying community stakeholder engagement across four country settings that largely mirror GPP goals and the GPP Blueprint, as well as highlighting challenges in the implementation of important guidelines. GPP guidance documents could be strengthened through greater focus on: identifying and addressing the community-specific roots of mistrust and its impact on trial literacy activities; achieving and evaluating representativeness in community stakeholder groups; and addressing the impact of power and funding streams on meaningful engagement and independent decision-making.

## Introduction

Stakeholder engagement, defined as processes through which those responsible for implementing trials build “transparent, meaningful, collaborative and mutually beneficial relationships” with “interested or affected” individuals or groups ([1],p.2), is increasingly recognized as foundational to ethically and scientifically rigorous HIV clinical trials. Previous shutdowns of trials to test new biomedical HIV prevention technologies as a result of concerted opposition from community stakeholders and civil society advocates [2–4] have stimulated action by global health and AIDS advocacy organizations to develop and disseminate guidelines for stakeholder engagement. In particular, UNAIDS/AIDS vaccine Advocacy Coalition (AVAC) Good Participatory Practice (GPP) Guidelines for Biomedical HIV Prevention Trials [1,5] and UNAIDS/WHO Ethical Considerations in Biomedical HIV Prevention Trials [6] establish core principles and processes to define and evaluate parameters for sound stakeholder interactions as well as rigorous HIV prevention trials. In addition, the GPP guidelines companion document, the GPP Blueprint for Stakeholder Engagement [7], provides a step-by-step guide for research teams to design a comprehensive stakeholder engagement plan.

Stakeholder engagement has become a mantra—among community advocates, civil society organizations (CSOs), clinical trialists, academic researchers and government sponsors alike [6,8]; however, further empirical research is needed to build on broad ethical recommendations, site reports, and studies conducted in the context of individual clinical trials in order to assess the implementation challenges for stakeholder engagement [9–12], in order to identify gaps between recommended best practices and implementation realities [13]. Given the urgent need for HIV-related clinical trials and the dramatic increase in the number of trials conducted, particularly in resource-limited settings [14], we aimed to advance an emergent social science of stakeholder engagement [15,16]. To that end, we conducted an embedded multiple case study in four international settings of previous as well as planned biomedical HIV prevention trials with the objective of eliciting the perspectives and experiences of a broad spectrum of respondents involved in and affected by community stakeholder engagement activities, and then comparing our findings with existing provisions from the cadre of GPP guidance documents. The purpose of this study was to assess and deepen the empirical foundation of the GPP guidelines and to highlight challenges in implementing these guidelines that may merit further attention in subsequent GPP iterations.

## Methods

**Fig 1:**
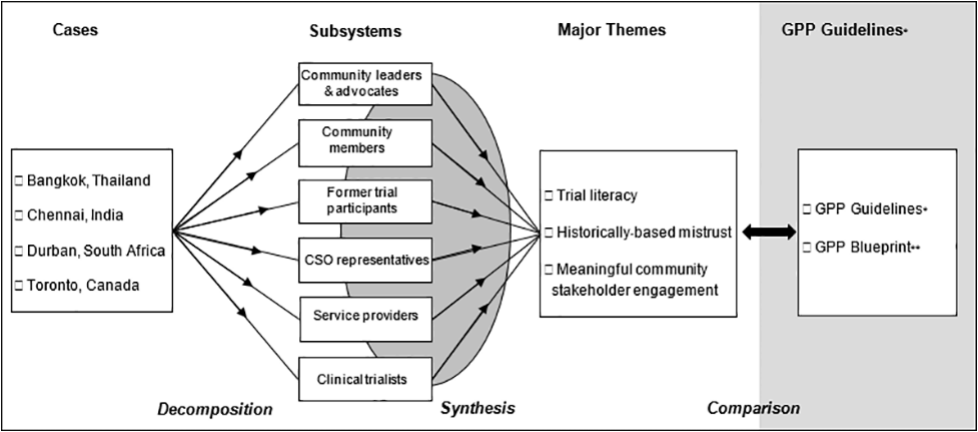
Conceptual model of multiple case study design. CSO: civil society organization, *Good Participatory Practice Guidelines for Biomedical HIV Prevention Trials [1]. **GPP Blueprint for Stakeholder Engagement (AVAC) [7].

From 2008–2012 we conducted an embedded, exploratory case study with a multiple case design. The case study approach is ideal for investigations that aim to move beyond narrow definitions of a research topic, address the context rather than isolated variables, and incorporate multiple sources of evidence [17]. We included multiple cases of the same phenomenon to enable us to compare and corroborate findings across cases [18,19]. While a holistic case study adopts one unit of analysis for each case (e.g., community stakeholder engagement in a particular trial), an embedded case study includes multiple units of analysis (e.g., community stakeholders, clinical trialists, CSO representatives) in each case [20] (Fig 1). The embedded approach improves validity through data source triangulation that includes a broad range of different stakeholder perspectives [21]. Our purpose in using a qualitative and, specifically, an embedded multiple case study approach was to achieve “analytical generalizability”: a single case design aims to assess testable propositions to understand a particular context, while a multiple case design aims to generalize analyses across settings, producing more robust findings with greater external validity [20]. We analyzed and synthesized findings across settings (i.e., multiple case design) in order to assess and expand GPP guidelines.

Each of the four country settings had been the site of previous HIV vaccine trials, the primary focus of this multiple case study. In Thailand, a Phase III prime/boost vaccine trial (RV144) was conducted from 2003–2009 with 16,402 volunteers aged 18–30 years, in a collaboration between the Thai Ministry of Public Health and the US Army Medical Research and US NIH/NIAID [22]. This was the first HIV vaccine trial to demonstrate protective efficacy, though the 31% reduction in HIV acquisition and waning efficacy was insufficient to support licensure [22]. The trial, centered 60 miles outside Bangkok in Chonburi and Rayong, involved 27 months of recruitment based on community (rather than individual) risk, extensive community education, engagement and monitoring activities, including visits to local villages and village heads, educational videos, ongoing assessment of social impact of participation (e.g., relationship problems) and follow-up of volunteers across the country [22,23].

In India, only three phase I HIV vaccine trials have been conducted, in 2003 [5,6,24], 2005 [8] and 2009 [9], with a total of 94 volunteers. The candidate vaccines were all safe and well tolerated, but did not warrant Phase 2 testing. In 2011, the Indian Ministry of Science and Technology partnered with the International AIDS Vaccine Initiative (IAVI) [3,24], which undertook consultations with multiple stakeholders, including meetings with representatives from government, scientists, nongovernmental organizations, and community-based organizations (CBO) serving men who have sex with men (MSM) in Chennai [4,24].

In South Africa, the HVTN 503/Phambili phase IIb test-of-concept study [25], aimed to test the Step Study vaccine in a region with a different dominant HIV clade. Phambili was launched in January 2007, designed to enroll 3000 predominantly heterosexual adults age 18–35 years across fives sites. In September 2007, after enrolling 801 participants, subsequent enrollment and vaccinations were stopped based on interim analyses of Step Study data, which met pre-specified futility criteria [26]. Unblinding of participants was begun in October 2007, with follow-up visits for safety monitoring and risk reduction counseling [25]. There was no evidence of vaccine efficacy. The South African AIDS Vaccine Initiative (SAAVI), in-country coordinator of HIV vaccine research, aimed (amongst others) to promote community education and mobilization, and social environments conducive to running HIV vaccine trials. SAAVI conducted extensive community stakeholder engagement activities at numerous clinical trial sites, as did sites themselves.

Canada was a site of the Step Study (HVTN 502), a multi-center, Phase II test-of-concept study with 3000 participants aged 18–45 years, enrolled from December 2004 to March 2007 [26]. The trial was funded by Merck and US NIH/NIAID. All further immunizations were halted in September 2007 when interim analyses met pre-specified futility criteria, with HIV infection rates no different or higher in the vaccine than the placebo arm of the study [26]. Subsequent analyses revealed time-limited increased susceptibility for HIV acquisition among a subset of vaccine recipients [27]. Community engagement focused on Toronto, the Canadian site of this multi-center trial, with public educational forums centered in the gay community and outreach through gay entertainment venues.

India and South Africa had additionally been sites of an earlier vaginal microbicide trial that was terminated early [28,29]. South Africa [30–32], Thailand [31–33] and Canada [34,35] have hosted several other biomedical HIV prevention trials, including trials of vaginal microbicides and pre-exposure prophylaxis (PrEP).

Lead organizations in each setting—University of Toronto, University of KwaZulu Natal, the Humsafar Trust, and VOICES-Thailand, jointly created and endorsed overall study objectives. Community and stakeholder collaborations within each setting involved: Mplus, SWING and Rainbow Sky Association of Thailand, Social Welfare Association for Men (SWAM) and Sahodaran in India, the South African AIDS Vaccine Initiative, and Maple Leaf Medical Clinic and AIDS Committee of Toronto. The lead organization in each setting in consultation with community collaborators determined participant subsystems and participants.

We used purposive sampling to recruit key informants (KIs) for in-depth interviews, informed by UNAIDS/AVAC GPP guidelines: “stakeholders” were those “individuals or collection of individuals who have a stake in a biomedical HIV prevention trial,” and “community stakeholders” as “individuals and groups that are ultimately representing the interests of people who would be recruited to or participate in a trial, and others locally affected by a trial” ([1],p.14). Further KI selection criteria were experience with HIV prevention and key populations within each setting, and inclusion of stakeholders across multiple trial-related subsystems in accordance with the embedded case design: CSO representatives, community leaders and advocates, service providers, clinical trialists, former trial participants, and community members from key populations at risk for HIV infection.

Focus group (FG) participants were recruited in collaboration with CBOs and community clinics serving key populations. In India, with a sustained, concentrated epidemic among MSM [36], FGs included MSM, male sex workers and MSM peer educators, with purposive sampling guided by inclusion of MSM with diverse sexual self-identifications [37]. In Canada, FGs reflected key populations: gay and other MSM, female sex workers, people of African and Caribbean descent, Aboriginal peoples, and people who inject drugs (PWID). FGs were not conducted in Thailand based on community collaborator input advising of challenges in discussing sensitive topics (i.e., HIV) and voicing critiques in a group; MSM, male sex workers, and PWID, key populations in Thailand [38], were included in individual interviews. In South Africa, with a generalized epidemic and extensive AIDS clinical trial experience, FGs were not conducted due to lack of funding; we conducted interviews with key community stakeholders.

We designed a semi-structured interview guide with open-ended questions and probes informed by a structured questionnaire previously developed for HIV vaccine trials [39], our team’s formative research [40], and earlier research on attitudes towards HIV vaccine trials [41,42] (see S1 File). Across sites, questions explored community stakeholders’ understanding and experiences of biomedical HIV prevention, clinical trials and key trial concepts (e.g., placebo, randomization, vaccine-induced seropositivity [VISP]); motivations for trial participation/engagement; trust in biomedical research; challenges in stakeholder engagement; and implications of early trial terminations or negative trial results. The interview guide did not present GPP guidelines. Interviews were conducted in local languages by trained research staff.

Interviews and FGs were recorded, transcribed in full, and translated into English. (The South African research team did selective transcription.) Data were analyzed in a multi-stage process. First, thematic content analysis [21,43] was used by within-country research teams to examine single case study data. Guided by the focal areas explored in the questionnaire, interviews were manually coded in English, including the use of line-by-line, open and axial coding, with openness to new themes that might emerge [44,45]. Second, after intra-case analyses, the Toronto team conducted cross-case analysis using qualitative meta-synthesis to compare and synthesize themes and sub-themes across settings, with triangulation of findings across settings to support validity [21]. Third, the principal investigator and research coordinator in each setting reviewed the multiple case findings to ensure they accurately reflected individual case study data and interpretations, and to verify the qualitative analysis [43]. We conducted peer debriefing with investigators and study coordinators, discussing the analysis and interpretations to support reflexivity [21,46]. Finally, after multiple case analysis, we explored and contrasted the case study findings with guidelines from UNAIDS/AVAC GPP [1] and the GPP Blueprint for Stakeholder Engagement [7]; we highlighted areas of alignment between our data and GPP guidelines as well as issues and challenges that appeared to be insufficiently addressed in current GPP provisions. Fig 1 depicts the overall research design.

The study received approval from the University of Toronto Research Ethics Board (REB), with administrative approvals provided by the directors of Mplus, Rainbow Sky Association of Thailand, and the Humsafar Trust in India, which did not have REBs. The South Africa study [47,48] and consent documents were approved by the Human and Social Sciences Ethics Committee of University of KwaZulu-Natal. All participants provided written informed consent, with all consent forms and information sheets approved by the University of Toronto REB. We identify participant quotations only by country setting and role to protect their anonymity.

## Results

We conducted 93 interviews and 21 FGs (n = 140) (N = 233) (Table 1); 65.6% (n = 153) were men, the majority (87.6%; n = 134) gay/MSM, 33.0% (n = 77) women, and 1.3% (n = 3) transgender women (TG). Interviews ranged from 45–60 minutes, and FGs from 1½-2 hours.

**Table 1:** Case study participants (and gender) by country and method (n = 233).

Country	Focus groups (n = 140)	Key informants (n = 93)
Thailand	-	42 (25 M, 14 W, 3 TG)
India	68 (68 M)	14 (14 M)
South Africa	-	14 (2 M, 12 W)
Canada	72 (29 M, 43 W)	23 (15 M, 8 W)

M: men

W: women

TG: transgender women

We identified three cross-cutting themes and seven subthemes supported by evidence from all four case studies (see S1 Table). Two additional subthemes were case-specific: “global disparities in resources” (India, South Africa, Thailand) and “early trial cessations” (Canada, South Africa).

### Theme 1: Trial Literacy

#### Communication challenges and trial-related misconceptions

Trial literacy emerged in all settings as a crucial component of community stakeholder engagement. Respondents across settings articulated ongoing challenges and complexities in communicating important scientific concepts to community stakeholders. Challenges were attributed in part to completely different vernacular vocabularies across multiple languages (even within country), low educational attainment among many key populations, belief in traditional healers, and lack of experience with research.

A Thai community advocate described a major component of this theme, indicating activities that promote trial literacy, such as clarifying key scientific terms and concepts, as incumbent on trial teams, and an essential precursor to invitations to participate: “At least tell us what it is, a vaccine, and then we can participate effectively, and be willing to support the trial. They must be willing to educate drug users to participate to know about the trial so they can make a decision” (PWID, KI, Thailand). A service provider from Canada articulated the importance of communication that acknowledges that various communities “have their own subculture” and the need to “speak the dialect of the population,” but described challenges in communicating about complex scientific issues: “We can talk about saturated fats and unsaturated fats; people can understand that. But the language for communicating about a vaccine is not in most people’s vocabulary” (African/Caribbean, KI, Canada). Service providers in India described lack of lay terminology to communicate “placebo” in Tamil. Peer educators resorted to analogies like “sugar water”, often failing to convey the scientific rationales behind placebo controls and double-blinding. Consequently, placebo was intuited by the local community as “deception” and “cheating”. An Indian KI explained that offering a placebo would be a deterrent to participation: “The community will not accept if we tell like this; even people who accepted [the invitation to participate] earlier will not accept, because they would be afraid that they are being cheated…they would not understand” (MSM community leader, KI, India).

Respondents across settings described challenges in encountering multiple misconceptions about key trial components. A common belief was that vaccines work by injecting a small dose of live virus: “they just put a teeny, teeny bit in your body….” (Aboriginal community, FG, Canada). A KI from Canada suggested this perception was common in their community: “There’s a lot of misconceptions about what a vaccine actually is; people will think, ‘Oh my god, you want to poke me with HIV to make me immune; you’re f—ing nuts’” (PWID, KI, Canada). A stakeholder from India similarly questioned the reliability of a vaccine containing deactivated virus: “You said dead virus is put in. How do we know…after going in it drinks blood and becomes alive?” (Kothi/MSM, FG, India).

Common misunderstandings about vaccines influenced conceptualizations of VISP, the indication, sometimes time-limited, of seropositivity as a normal immune response to HIV vaccination [49]. Across settings, community stakeholders expressed dismay about possible VISP, suggesting that if they tried to explain it to local communities it would deter trial participation: “They will get real scared and confused and run away” (MSM peer educator, KI, India); “Try to explain this to somebody who speaks Cree or Ojibwa, and there aren’t words to describe this” (Aboriginal peer educator, FG, Canada). A transgender peer educator from Thailand explained, “When I first heard that my blood would become positive after I’m vaccinated, I wondered if my body would be able to prevent HIV” (TG, KI, Thailand).

Community members were seen as trying to make sense of complex scientific information about vaccines and clinical trials by drawing on existing knowledge or experience. A Thailand KI described challenges in communicating about a vaccine trial in that “people still have old beliefs…some communities have heard about herbal medication for HIV that costs only 800 Thai baht [~$25 USD]; if it doesn’t work, they will get lifetime healthcare for free. So we need to explain to them that our vaccine is not like that….” (Clinical trialist, KI, Thailand). A South Africa KI explained: “People take the little snippet of information they’re given and they wrap it into their local belief systems…and from there try to make sense of it. And it’s not surprising that there are just incredible amounts of misunderstanding how it works” (CSO rep., KI, South Africa). Thus, KIs and FG participants reported a broad range of trial-related misconceptions and communication gaps about key vaccine, clinical trial- and HIV-related concepts, and highlighted difficulties that emerge in trial literacy activities.

#### Preventive misconception

Misunderstandings about the nature of clinical trials, placebo-controls, randomization, and conflation of HIV prevention trials with prevention programs were identified, in particular, as supporting preventive misconception and risks for behavioral disinhibition. Preventive misconception (analogous to “therapeutic misconception”, i.e., “failure to appreciate the difference between research and treatment” in clinical trials [50]) is the tendency for participants in prophylactic clinical trials to overestimate the probability of being assigned to the experimental versus control group and/or to assume that the experimental drug or vaccine being tested is effective [37,51].

Some respondents feared their communities would perceive a trial vaccine to confer 100% protection. As a Canadian KI explained, “The understanding of vaccine to the general public means I am immune: you have given me the invisible cloak; you’ve given me the Superman suit. I’m all good” (African/Caribbean, KI, Canada). A KI from India explained, “[Trial participants] may not know much information about placebo. They will believe that ‘I have been given an HIV vaccine, I can do whatever I want” (MSM peer educator, KI, India). Frequent confusion was evinced across settings with community stakeholders alternating between talking about “vaccine” and “cure,” “trial” and “program,” demonstrating conflation of HIV prevention and treatment trials, and of trials and interventions. A Thailand KI reported in reference to an HIV vaccine trial, “Some people get confused that this vaccine works as a cure,” and further, that “something new that they find out about comes in the form of hope, too” (Service provider, KI, Thailand). An India KI reasoned that researchers wouldn’t invest time and money in testing a candidate vaccine unless they already knew it worked: “If it is not working, then why would they be actually testing it?,” (MSM peer educator, KI, India) reflecting a significant disjoint in understanding between researchers and community stakeholders indicative of preventive misconception.

### Theme 2: Challenges Posed by Historical Mistrust

#### Histories of colonialism and exploitation

Conceptualizations of clinical trials and biomedicine were described in the context of historical experiences with colonialism, marginalization, and exploitation in each of the case settings. In Canada, an Aboriginal community stakeholder revealed not lack of understanding but historically-based mistrust as a primary challenge to stakeholder engagement: “Our people don’t trust the government anymore because we’ve been cheated so many times. The Whites brought polio to our people, other diseases. It’s the government telling us, ‘take it, it will not hurt you’” (Aboriginal peer educator, FG, Canada). Analogously, in South Africa a community stakeholder voiced concerns rooted in the historical oppression and exploitation of Africans: “You have people who have the perceptions that these trials are not meeting ethical standards, that people are being harmed, people’s rights are not being adequately respected—the notion that these trials are happening in Africa and Africans are being used as guinea pigs” (CSO rep., KI, South Africa). In both India and Thailand, concerns emerged around why trials needed to be conducted in their countries, if safety protocols were being followed, and if testing was also being conducted in rich countries. An India KI reported, “I even doubt whether a Phase I trial [in India] among normal human volunteers was actually conducted” (Service provider, KI, India).

Community stakeholders across key populations at risk for HIV and across settings interpreted their being recruited for trials due to their high risk as unethical:

It’s almost inherent in the study that in order for them to be able to really study the effects of the vaccine, they really, in essence, want you to contract HIV and that’s why you’re chosen for the study, because you’re in a high-risk group for HIV infection. And so a small voice in my head is saying, well, this is kind of f—d up. (Former trial participant, KI, Canada)

In South Africa, certain community members reportedly viewed researchers with suspicion, a view potentially intensified by negative trial results, as “playing with participants’ lives.”

Community respondents described experiential knowledge through face-to-face communication, including testimonials from former trial participants, as crucial elements of building trust with researchers. An Indian MSM recommended, “Let them say that, ‘We also volunteered like you. We did not have any problem.’ Like that, if they give us 100% confidence, they [MSM] will come definitely” (Kothi/MSM, FG, India). An Aboriginal stakeholder reported, “I will wait to see the regular people who come in for two years to take the vaccinations; and then I’d see if any of them drop dead or grow buffalo humps…then I might…start considering it after that” (Aboriginal gay man, FG, Canada). A South African respondent articulated the need for ongoing communication as key to offsetting mistrust: “let people know how things are going, and if there is a problem, be honest” (CSO rep., KI, South Africa).

#### Global disparities in resources

Disparities between trialists/sponsors and trial sites emerged as a significant element of mistrust, with a common construal of low-income country participants being used as “guinea pigs” to benefit high-income countries. An Indian service provider asserted that Indians were being used as “guinea pigs” and transposed concerns rooted in colonialism to present day economic disparities: “Most of these trials are deliberately conducted among people who are economically disadvantaged and who are from developing countries” (Service provider, KI, India). A South Africa CSO representative described community-based concerns that “Africans are being used as guinea pigs.” A Thailand community advocate wanted evidence of trials abroad before local implementation: “We would watch and see someone else first, especially…if people in Washington or New York are being vaccinated or not. If they are…not just the black people or others, then…if it is the nice looking university students, then, OK…probably OK” (CSO sex worker, KI, Thailand). An India community leader was similarly curious about “whether trials are happening in other countries?…Why does this need to be conducted among MSM in India…?” (MSM, KI, India).

#### Early trial cessations

Respondents in Canada and South Africa, sites of the Step Study and Phambili trials, respectively, in which vaccinations and enrollment were suspended due to unforeseen risks to some vaccine recipients [26,27], indicated associations between trust and how early trial cessations are managed. A Canadian community advocate explained how past history can be connected with current experiences of trial closures: “They [PWID] hate them [medical providers]; they feel persecuted by them; they feel belittled and judged, and I don’t blame them because they are…. There has to be some repair done. I think they’ve widened the gap of mistrust. I might feel like I was lied to” (PWID, KI, Canada). A former trial participant expressed concern in how he had received information about the trial cessation and possible increased susceptibility:

The way I found out about this was through the media so initially there was some hostility on my part because I felt well, I’m in this study, if I’m finding out through the media that means that someone knew a week ago at least, you know what I mean? It’s very scary when you find out about something that you’re involved in, not from the people that you were working with but from an outside source. (Former trial participant, KI, Canada)

A South African researcher highlighted the need for trialists to clearly explain the chance of early trial termination, stating “People get really psyched up and invested in a trial”, while also admitting, “I don’t think we communicate that possibility as well as we need to” (Researcher, KI, South Africa). A former participant’s experience embodied this researcher’s concern, further revealing mistrust: “They never actually said that anything like this could possibly happen, but of course if they did nobody would take the trial” (Former trial participant, KI, Canada).

### Theme 3: Meaningful Community Stakeholder Engagement

#### Early engagement

Respondents across case studies highlighted the importance of engaging community stakeholders early in trial planning processes. A South African CSO representative stated, “An ideal model of community engagement would involve communities during protocol formulation stages to determine the community’s perceptions of the social value of the research.” Early engagement was articulated as an important avenue to work through historically-based mistrust, a mechanism through which community stakeholders would feel a greater sense of control and ownership in contributing to the research endeavor. Respondents across settings endorsed early involvement as a key component of achieving meaningful engagement.

Communities need to be engaged more over the life of a trial: They should not only be engaged during community meetings when trials are recruiting and then again when results are going to be announced: this does not constitute meaningful community involvement. (CBO rep., KI, South Africa)

Community stakeholders from Canada, while indicating the importance of early engagement, expressed caution as to how it is implemented. An Aboriginal peer educator advised the importance of engaging informal leaders and community Elders, but articulated challenges in knowing who to approach: “And it seems like decisions are getting made in the Chief Council, but it’s some little old lady cooking soup in the back of the center who actually everyone checks in with first”; and further that “outsiders”, including HIV experts and leaders, should go through Aboriginal political leaders rather than approach Elders directly: “You can’t really say, ‘I’m 30-years old; okay, Elders, come get educated by me’; I mean, that’s not respectful” (Aboriginal community, FG, Canada).

#### The breadth of “community” representation

A challenge to community stakeholder engagement was expressed in terms of overly narrow conceptualizations of who constitutes local community. A Canadian community stakeholder stressed, “the whole community, everyone should be involved”, including different ethnic/racial and socioeconomic backgrounds: “That way it’s being tested so that all populations can say this is something that is correct data” (Female sex worker, KI, Canada). Yet community stakeholders described that recruitment and consultation across a broad spectrum of community members is limited. An Indian stakeholder lamented that consultative processes were limited to individuals with existing CBO linkages: “Since I am working in a CBO, [trialists] invited me [for a consultation meeting]; others thus do not know about this and could not participate” (MSM community rep., KI, India). A South Africa CSO representative expressed concerns with “site hand-picking” of community members (CSO rep., KI, South Africa). In particular, a South Africa KI explained that community advocates are often distanced from trial involvement, approached as an afterthought, and constrained to narrowly proscribed roles: “Advocates are seen as the necessary noise that should come after the agenda has been drawn, instead of being the ones drawing some of the agenda because they are so much in touch with communities” (CBO rep., KI, South Africa).

#### Clear and appropriate roles

CSO representatives articulated the importance of tailoring roles and responsibilities to people’s skills, capacities and preferences, and challenges and uneven implementation of this recommendation. A community member felt that involvement should be comprehensive, saying “My community wants to feel empowered; they want to feel engaged. Engage them in the actual setting up of the trial, in recruiting for the trial. Include them in every aspect” (PWID, KI, Canada). Yet a South Africa CSO representative explained that involving everyone in every step of the planning process would not necessarily lead to positive outcomes: “If you have people at the table not because they are going to contribute in work but because they just want to be there, then you are going to expand the amount of work that needs to get done and the complexity of it, but not necessarily improve the outcome” (CSO rep., KI, South Africa).

CSO and other stakeholders articulated specific roles for advocacy organizations and community advisory boards (CABs) that drew on their existing strengths. Several KIs explained that advocates play a particularly important role in explaining scientific results to community stakeholders and acting as a bridge to biomedical stakeholders, even more so when a trial is terminated early:

So, when things get difficult and you need friends; or you have a hard time like when a trial closes early; you already have people who have a relationship with you, who are aware of your intentions; can be the voice in the community and with the media and with other stakeholders to help get the message out and do some damage control. (CSO rep., KI, South Africa)

However, community advocates perceived that their effectiveness in this role was constrained by unequal power dynamics and limited communication with research teams. An Indian CBO leader expressed frustration about abrupt cessation in communication from researchers regarding a planned vaccine trial that was terminated after the Step Study cessation, explaining that long-term credibility and trust with research stakeholders was jeopardized:

No proper information was given why they were no longer calling us… A meeting that was scheduled was cancelled. I could not face my community people when I had already spread messages about the trial coming. They were asking, ‘What is happening now? You asked us to come for meetings and now no noise’. I was very angry at that time. I felt as though we were being used–like a ‘use-and-throw’ [disposable object].” (MSM CBO rep, KI, India)

This commentary also typifies the need to address unequal power dynamics between CBOs and research teams. CBO representatives sought assurances that they would be consistently supported and engaged rather than simply “used” to recruit participants.

KIs asserted that while CABs may serve key functions in bridging community and researchers, they often lack sufficient leverage and control within the research team to insist on certain priorities, particularly when they are beholden to research stakeholders for their funding. A South African KI questioned the extent to which CABs could operate autonomously from research teams: “I also think the CAB’s being constituted by the research community and being sustained by the research community introduces some bias to what they do” (CSO rep., KI, South Africa). Another South African KI highlighted the differing capacities of CABs to assert their own priorities: “I’ve seen many CABs, not all CABs, being empowered enough to engage actively in the research” (CSO rep, KI, South Africa).

#### The benefits of engagement

Benefits of meaningful community stakeholder engagement were articulated by CSO representatives and advocates, who described wanting to contribute to ethical oversight of trials that contribute to their communities, and by community stakeholders, who reported feeling themselves a part of a team with a common goal in a shared research endeavor. Some former Step Study volunteers in Canada expressed positive feelings despite early trial termination: “I felt a bit like a pioneer…I was actually quite proud to be part of something that could have some far-reaching impact” (Former trial participant, KI, Canada). Another Canadian volunteer echoed these sentiments, saying they felt they were “an important part of the whole team”: “They didn’t just treat me like a patient or a research study participant. I felt like I was an important part of the whole process. I wasn’t just a guinea pig” (Former trial participant, KI, Canada). A Thailand trialist similarly described the camaraderie developed among some volunteers: “Now we are having a club in our trial for our participants who want to create some kind of HIV/AIDS campaign in the future” (Clinical trialist, KI, Thailand). CSO representatives in South Africa asserted that meaningful engagement can help to offset potential mistrust associated with negative trial results: “when a trial closes early, you already have people who have a relationship with you to help get the message out” (CSO rep., KI, South Africa).

## Discussion

Across embedded case studies conducted in four country sites of previous HIV vaccine and other biomedical HIV prevention trials, we identified shared priorities and concerns about community stakeholder engagement. We also identified challenges to the implementation of stakeholder engagement: lexicon challenges and misconceptions that imperil sound communication; legacies of historical exploitation that jeopardize trust; and disparities in power, resources, and scientific knowledge that impede equitable and mutually beneficial relationships with shared power and decision-making. In applying and juxtaposing these themes from the multiple case study to explore GPP guidelines, we discerned congruencies between the multiple case study results and GPP principles and practices, as well as complexities and challenges for the implementation of GPP guidelines and recommendations for community stakeholder engagement in diverse global settings.

### Trial Literacy

Trial literacy was a pervasive concern that emerged across cases. GPP guidelines [1] directly address trial literacy in advocating “strategies to be used to ensure comprehension of critical trial-related terms and concepts” [1] and observing that trial literacy is crucial for ethically valid informed consent [1,5]. Trial literacy is further articulated in GPP guidelines as a mechanism to offset power imbalances between research teams and other stakeholders, including participants and participating community. To this end, the GPP Blueprint asks researchers to assess how knowledgeable CAB and local community members are about the clinical research process, HIV prevention, HIV prevention research, and “the science involved in the proposed research” ([7],p.6).

Our findings, while corroborating the importance of trial literacy, suggest additional complexities in implementation. Low levels of scientific literacy and historically-based mistrust emerged as dynamic and cumulative, rather than as independent or merely co-existing factors. Although GPP guidelines acknowledge that engagement processes may be “overlapping” ([1],p.26), the guidelines might benefit from more explicitly drawing attention to the reciprocal impact of low scientific literacy and mistrust. Community stakeholders may be predisposed to suspicion about biomedical HIV prevention trials based on past negative encounters with medical research and historically-rooted mistrust in government; misunderstandings of the science underlying clinical trials (i.e., placebo, random assignment, VISP) may be superimposed on this distrust thereby engendering perceptions that trial literacy activities are not adequately or transparently implemented. This corresponds with evidence of not only misunderstanding, but of non-acceptance of some of the information offered by peer educators and trialists [52]. Thus while inculcating trial literacy is an important goal, which may in turn facilitate some equalization of power imbalances as noted in GPP, the very power imbalances to be addressed may engender doubt and disbelief in both the message and the messenger. Planned and strategic efforts to engage community stakeholders and build trust may be fundamental to effective trial literacy activities [53]. Attention to interconnections between pre-existing perceptions of research among community stakeholders and current trial activities is key, as is continued re-evaluation of these perceptions in light of likely shifts over time [7].

### Mistrust

Various permutations of historically-based mistrust emerged in each setting, with reference to colonialism, exploitation and marginalization. GPP guidelines directly address mistrust, recommending that trial teams undertake formative research to understand “power dynamics, local perceptions, channels of communication and decision-making, and local history of research, as well as the needs and priorities of people who are locally affected by and able to influence the trial” in order to build more trusting interactions at the researcher-community interface [1]. The GPP Blueprint instructs research teams to consider what kind of HIV-related work and activities have taken place at the trial site during intervals between trials, how community stakeholders and the CAB have responded to past engagement efforts ([7],p.5), and to list any “attitudes, beliefs, or sociobehavioral factors in the local community that could interfere with recruitment or trial conduct (e.g. social stigma, religious and traditional beliefs or practices, gender discrimination, misconceptions about research, mistrust of research and researchers)” ([7],p.9).

The various case study narratives illustrating mistrust and its impact on community stakeholder engagement indicate that acceptable and effective engagement is likely to adopt a variety of configurations across settings, with no universally applicable template [10,54–56]. Evidence from this multiple case study suggests that overly simplistic models and “prefabricated” efforts based on “tick-box” approaches to stakeholder engagement [10] are unlikely to be effective because they are not well tailored to the particular historical context, experiences and (mis)conceptions about research(ers) prevailing in the participating community [57–59]. This demonstrates an important distinction between community involvement as an end unto itself and community stakeholder engagement as a dynamic process that aims for collaborative, respectful, responsive and, ideally, sustained interactions with the goal of advancing more ethically rigorous clinical trials.

### Early Trial Cessations

Findings from Canada and South Africa cases, sites of the Step and Phambili trials that were halted, illustrate that early trial cessations have the potential to build on underlying mistrust and thereby reinforce perceptions that research has undisclosed harmful effects on community stakeholders—even more so if these stakeholders perceive that potential risks were not adequately explained. Our findings corroborate GPP recommendations for early and careful planning for unexpected or negative developments in order to prepare for crises and controversy that may arise in clinical trials [1], building on earlier recommendations for discussion and dialogue to “minimize the risk of misinformation” when a trial is stopped early or unexpectedly [5]. Importantly, stakeholder narratives in these two cases demonstrated that the goals of community stakeholder engagement may shift over time; and early engagement can serve as a proactive strategy to mitigate misperceptions and address mistrust that may otherwise intensify fallout from negative trial results [60]. This theme clearly intersected with the “benefits of engagement”, which underscores GPP’s assertion that sound engagement should lead to relationships perceived as mutually beneficial, collaborative and supportive of rigorous trials [1]. Meaningful engagement also may mitigate negative fallout from unexpected trial cessations and disappointment when products do not demonstrate efficacy. Although some former trial participants indicated disillusionment and mistrust, others specifically demonstrated understanding that the negative outcomes were unforeseen, and expressed feeling that they were nevertheless proud to be a part of a team effort to advance science and give back to their communities. Some CSO representatives perceived sound prior engagement as a kind of “anesthetic” for negative trial results.

### Early Engagement and Breadth of Community

Case study findings across settings, while evoking the importance of early engagement and broad representation from the participating community, indicated implementation challenges that have the potential to subvert the goals of these important processes. GPP recommends a range of practices to operationalize meaningful community stakeholder engagement: researching the participating community, establishing community advisory mechanisms, developing plans for engagement and capacity-building, communications and issues-management, and sustaining engagement across trials [1]. The GPP Blueprint provides extensive worksheets to guide these tasks, encouraging researchers to assess the sociocultural landscape of the trial community, identify and prioritize potential stakeholders, and create a comprehensive stakeholder engagement plan [7].

Community stakeholder narratives across cases revealed that groundwork with populations among those most gravely impacted by HIV and simultaneously impacted by colonialism and exploitation in a given context (e.g., Aboriginal peoples in Canada, Black South Africans) may encounter particular challenges in important processes of engaging early and with a broad array of community representatives. Respondents from Canada indicated that researchers’ well-intentioned entreaties to engage Aboriginal communities may be perceived as disrespectful or suspect to the extent they do not employ culturally appropriately means of initial engagement; and, further, that knowing whom to approach is unlikely to be apparent to an “outsider” [61]. The legacy of Apartheid emerged in South African CSOs’ discussion of the potential for historically-based mistrust by Black South Africans of medical research led by predominantly white researchers—invoked in the frequent ‘guinea-pig’ metaphor. Approaches to addressing these challenges may benefit from specific knowledge of the organizations and individuals understood to be culturally accepted gatekeepers, and the community’s culturally sanctioned mechanisms for initiating communication in each context [61]. Actively involving social scientists who have existing familiarity with the local historical and social context of the research site and participating community, or who are funded to conduct in-depth investigation to study and describe the local context [57], may be a valuable asset to community stakeholder engagement. This might specifically include action research processes that involve social scientists as part of engagement teams to inform strategies for meaningful engagement. Engagement strategies should be informed by local advisors, and periodically evaluated for outcomes [62]. Otherwise well-intended overtures to engage particular communities may meet with obstacles, well beyond the scope of any particular clinical trial, that render generic ‘good practices’ ineffective.

A related implementation challenge emerged in concerns about the representativeness of community stakeholders and the breadth of engagement activities. Cross-cutting themes indicated that in circumstances in which community engagement is undertaken without careful consideration of who should be engaged, through which strategies, and their actual role, engagement risks being unwieldy or a tokenistic effort to support a politically correct façade. Although it was deemed important, in principle, that CABs and other community stakeholder groups should represent the broader participating community, in practice, respondents indicated that recruitment and consultation activities are sometimes limited to previously known individuals with existing ties to prominent CBOs. This can result in situations in which individuals outside of CBO infrastructures are largely excluded from community stakeholder engagement activities.

Previous efforts to lend clarity and uniformity to the definition of “stakeholder” in community stakeholder engagement activities in clinical trials indicate the difficulties in this endeavor and help to articulate the various layers of stakeholders that might be appropriately involved [5,54,63–65]. Other investigations have found that “discerning the community of stakeholders was not a linear process, but rather a complex and recursive set of activities” [57] and that choosing representatives “that would be accepted by the local community” remains a significant challenge [66].

The GPP guidelines highlight the importance of an “inclusive perspective for identification of potential stakeholders” ([1],p.16) to help determine “which groups or individuals are relevant stakeholders and why” ([1],p.31) and the GPP Blueprint asks researchers to question, “How well does the current CAB composition reflect the community population?” ([7],p.6). However, while some degree of flexibility is probably desirable, these guidelines do not address how an external research team should assess the representativeness of the CAB or other existing stakeholder groups to the wider community [62]. Furthermore, broader questions exist as to when community engagement is warranted or may be an ethical requirement for research; these may benefit from ethics board involvement, although few guidelines presently exist [62,67]. In fact, community engagement activities themselves may raise additional ethical challenges requiring ethics committee oversight [68]. In practice, as described in the current investigation, research teams are often dependent upon a relatively small number of community members who have prior experience and interest in working with clinical trials. Given the importance of forming community stakeholder groups that are representative of the wider community, and the discretionary power of stakeholder groups to influence trial activities, further attention should be paid in future GPP iterations to this significant implementation challenge.

### Global Disparities in Resources

A final challenge that arose in the implementation of community stakeholder activities concerns the impact of global disparities in wealth and power. GPP states that CABs should strive for independence even while they are funded by trials [1]. The GPP Blueprint directs researchers to “list any economic or structural issues (e.g. poverty, lack of education, unemployment) that may interfere with recruitment, adherence, or other aspects of the trial” ([7],p.11). Yet the acknowledgment of poverty and low educational attainment in a study site is only an initial step in addressing the impact of structural inequalities between researchers and sponsors from high-income countries and trial sites in resource-limited settings. Knowledge alone is unlikely to offset such structural inequities.

GPP further stipulates that stakeholders and advisory mechanisms (including CABs) not be directly involved in trial activities such as recruitment [1]; however, our findings indicate this blanket assertion, as well as the need for CABs to exercise independent decision-making, elides tensions and power dynamics in practice. The degree to which this recommendation is enacted in practice should be explicitly investigated at trial sites across settings; it is likely that a variety of different approaches are implemented with varying levels of success.

Although bidirectional communication between research stakeholders and community stakeholders is a valued goal, communications are often undergirded by funding streams that operate as a unidirectional pipeline. It is beyond the scope of HIV prevention trials to level global economic disparities, however, it is incumbent on stakeholders from high-income countries to address and mitigate the impact of underlying structural inequalities on power differentials within the trial team and on the independence of community advisory mechanisms. Guidelines that articulate how to navigate the CAB’s and other community representatives’ dependency on international funders associated with a trial without compromising their mandate to represent the best interests of local communities may help to mitigate the deleterious effects of economic disparities on meaningful community stakeholder engagement. Structural interventions, such as diversification of trial funding sources and sustained funding for community advisory mechanisms outside of particular trials, may be an important mechanism to help remedy the impact of disparities in resources on trial teams and community oversight.

Differences emerged among stakeholder narratives within case study settings in regard to discussion of global economic disparities. Community stakeholders, particularly community advocates and CBO leaders, articulated the challenges that ensue from unequal power and decision-making in the context of international HIV prevention trials more so than did CSO leaders or trial staff, or members of key populations. Community stakeholders more broadly, in contrast to research stakeholders, also raised the importance of appropriate roles to meaningful engagement. These differences highlight the importance of defining and identifying community stakeholders among the broader definition of stakeholders. Differences also emerged across case study settings, which contribute to illustrating the impact of global disparities in resources on community stakeholder engagement: these issues arose only in resource-limited settings, more likely to experience disparities with high-income country researchers and funders, not in Canada.

### Limitations

Our findings should be interpreted in light of study limitations, including the lack of uniformity inherent in an international, multiple case study: the level of exposure to biomedical HIV prevention trials, the participant subsystems, and translations in lay language to describe clinical terminology varied in each setting. The qualitative approach suggests caution in generalizing findings to other settings. As an embedded, multiple case study, we prioritized participant subsystems and key populations deemed appropriate and feasible by research teams and community collaborators in each setting to facilitate our objective of achieving analytical generalizability through data reflecting local perspectives and experiences [20]. Nevertheless, each setting had hosted trials of HIV vaccines and at least one other biomedical HIV prevention technology (i.e., vaginal microbicide or PrEP). The embedded case design, with triangulation of data from respondents representing multiple stakeholder subsystems within each setting, and multiple cases with evidence supporting common themes derived inductively across settings, support the validity of the results [46]; further investigations will need to evaluate prevalence and generalizability to other sites and populations.

## Conclusions

Themes from across multiple settings broadly support the importance of the UNAIDS/AVAC GPP guidelines and the GPP Blueprint for Stakeholder Engagement, both of which emphasize the complexities underpinning trials, such as vulnerable participants, mistrust, and power imbalances, and endorse practices to build stakeholder relations—such as engaging early, being broad and inclusive in representation, and being clear about roles [1]. We identified cross-cutting and site-specific barriers and enablers, as perceived by diverse stakeholders in four country settings, which contributes to emerging scholarship on community stakeholder engagement. Overall, these findings underscore both the importance of investment in community stakeholder engagement and the complexity of such investment; and they contribute to an emerging database of community stakeholder engagement experiences and practices that can be used to inform engagement recommendations for the development and testing of sorely needed new HIV prevention technologies for key populations globally.

## Supporting Information

S1 File Semi-structured interview guide. (DOCX)

S1 Table Multiple case study themes, subthemes and quotations. (DOC)
