# Supplementary material for: Towards a Science of Community Stakeholder Engagement in Biomedical HIV Prevention Trials: An Embedded Four-Country Case Study
Source: PLoS One. 2015 Aug 21;10(8):e0135937. doi: 10.1371/journal.pone.0135937 (PMC4546590; doi:10.1371/journal.pone.0135937)
Supplement: S1 File — (DOCX) [file pone.0135937.s001.docx]

**S1 File. Semi-structured interview guide.**

1. What is your interest and/or involvement in HIV prevention trials?
2. What is/has been your involvement in HIV vaccine trials (or HIV prevention trials)?
   - What is your role/ what contribution do you make?
   - In what ways do you think your contribution could be different?

[*For trial participants and participating communities*]

2a. What is your understanding of an HIV vaccine (or HIV prevention) trial?

- What are some of the positive aspects?
- What are some of your concerns?
- Explore key terminology: placebo, randomization, vaccine-induced seropositivity (VISP)

1. What do you think the impact of HIV prevention trials has been?
   - On different stakeholders (and the relationships between them)?
   - What are some of the positive impacts?
   - What are some of the negative/ complicating impacts?
   - Can you provide examples/ specific stories [for the above]?

1. To what extend have community stakeholders been involved in HIV prevention trials?

- How have they been involved?
  - Can you provide examples/ specific stories [for the above]?

1. Some HIV prevention trials have been suspended (that is there is no more enrolment or immunization).
   - Can you tell us what you know about this?
   - Can you tell us what you know about the STEP and Phambili trials?
   - Can you tell us what you know about other trials?
   - What was your response to this news?
   - What have you heard about the response of others?
2. What do you think has been the impact of suspending the Phambili/ STEP trials? [If *participant is not aware of this, then* “what might the effect be?”]
   - On various stakeholders
   - On relationships between various stakeholders (e.g. researchers and communities; researchers and government; researchers and RECs; researchers and advocates)
   - What were the attitudes of various stakeholders to the trial closure?
   - What worked well in terms of this trial?
   - How might have it been handled differently?
   - How might the developments in STEP/Phambili impact on future trials?
3. How could stakeholders be better engaged in HIV prevention trials and HIV vaccine trials?
   - Specifically, how could civil society be better engaged in HIV prevention trials and HIV vaccine trials? Community groups? Other stakeholders?
   - Can you tell us any stories of successful civil society involvement; community engagement?
