# Supplementary material for: Towards a Science of Community Stakeholder Engagement in Biomedical HIV Prevention Trials: An Embedded Four-Country Case Study
Source: PLoS One. 2015 Aug 21;10(8):e0135937. doi: 10.1371/journal.pone.0135937 (PMC4546590; doi:10.1371/journal.pone.0135937)
Supplement: S1 Table — (DOC) [file pone.0135937.s002.doc]

**S1 Table. Multiple case study themes, subthemes and quotations.**

| **Theme 1. Trial Literacy** |
| --- |
| ***Communication challenges and trial-related misconceptions***  At least tell us what it is, a vaccine, and then we can participate effectively, and be willing to support the trial. They must be willing to educate drug users to participate to know about the trial so they can make a decision (PWID, KI, Thailand)  We can talk about saturated fats and unsaturated fats; people can understand that. But the language for communicating about a vaccine is not in most people’s vocabulary (African/Caribbean, service provider, KI, Canada)  The community will not accept if we tell like this; even people who accepted [the invitation to participate] earlier will not accept, because they would be afraid that they are being cheated…they would not understand (MSM community leader, KI, India)  They just put a teeny, teeny bit in your body…. (Aboriginal community, FG, Canada)  There’s a lot of misconceptions about what a vaccine actually is; people will think, ‘Oh my god, you want to poke me with HIV to make me immune; you’re f--ing nuts’ (PWID, KI, Canada)  You said dead virus is put in. How do we know...after going in it drinks blood and becomes alive? (Kothi/MSM, FG, India)  They will get real scared and confused and run away (MSM peer educator, KI, India)  Try to explain this to somebody who speaks Cree or Ojibwa, and there aren’t words to describe this (Aboriginal peer educator, FG, Canada)  When I first heard that my blood would become positive after I’m vaccinated, I wondered if my body would be able to prevent HIV (TG, KI, Thailand)  People still have old beliefs…some communities have heard about herbal medication for HIV that costs only 800 Thai baht [~$30 USD]; if it doesn’t work, they will get lifetime healthcare for free. So we need to explain to them that our vaccine is not like that…. (Clinical trialist, KI, Thailand)  People take the little snippet of information they’re given and they wrap it into their local belief systems...and from there try to make sense of it. And it’s not surprising that there are just incredible amounts of misunderstanding how it works (CSO rep., KI, South Africa)  Research literacy is absolutely important and the job is on civil society groups to do it; because when it’s done by the researchers, it looks like social marketing and all you want to do is make sure people accept your research, rather than coming from a neutral perspective (CSO rep., KI, South Africa)  One big area where there’s still a lot of gap is research literacy within the general communities (CSO rep., KI, South Africa) |
| ***Preventive misconception***  The understanding of vaccine to the general public means I am immune: you have given me the invisible cloak; you’ve given me the Superman suit. I’m all good (African/Caribbean, KI, Canada)  [Trial participants] may not know much information about placebo. They will believe that ‘I have been given an HIV vaccine, I can do whatever I want (MSM peer educator, KI, India)  Some people get confused that this vaccine works as a cure; something new that they find out about comes in the form of hope, too (Service provider, KI, Thailand)  If it is not working, then why would they be actually testing it? (MSM peer educator, KI, India) |
| **Theme 2. Challenges Posed by Historical Mistrust** |
| ***Histories of colonialism and exploitation***  Our people don’t trust the government anymore because we’ve been cheated so many times. The Whites brought polio to our people, other diseases. It’s the government telling us, ‘take it, it will not hurt you’ (Aboriginal community rep., FG, Canada)  You have people who have the perception that these trials are not meeting ethical standards, that people are being harmed; people’s rights are not being adequately respected—the notion that these trials are happening in Africa and Africans are being used as guinea pigs (CSO rep., KI, South Africa)  I even doubt whether a Phase I trial [in India] among normal human volunteers was actually conducted (Service provider, KI, India)  It’s almost inherent in the study that in order for them to be able to really study the effects of the vaccine, they really, in essence, want you to contract HIV and that’s why you’re chosen for the study, because you’re in a high-risk group for HIV infection. And so a small voice in my head is saying, well, this is kind of f--d up (Former trial participant, KI, Canada)  Let them say that, ‘We also volunteered like you. We did not have any problem.’ Like that, if they give us 100-percent confidence, they [MSM] will come definitely (Kothi/MSM, FG, India)  I will wait to see the regular people who come in for two years to take the vaccinations; and then I’d see if any of them drop dead or grow buffalo humps…then I might…start considering it after that (Aboriginal gay man, FG, Canada)  Let people know how things are going, and if there is a problem, be honest (CSO rep., KI, South Africa)  For the larger community and civil society groups within them, with limited information, this stuff is very taxing, and it fuels all these ideas of guinea pigs in our country, and people coming in to do research and we are not getting anything out of it, and our women are getting infected. There is some of that sentiment too, and that is often because they haven’t been given a lot of information or don’t have a good understanding (CSO rep., KI, South Africa) |
| ***Global disparities in resources***  Most of these trials are deliberately conducted among people who are economically disadvantaged and who are from developing countries (Service provider, KI, India)  The notion that these trials are happening in Africa, and Africans are being used as guinea pigs (CSO rep., KI, South Africa)  We would watch and see someone else first, especially… if people in Washington or New York are being vaccinated or not (laughing). If they are, that is not just the black people or others then…if it is the nice face looking university students, then, Oh ok… probably ok (CSO sex worker, KI, Thailand)  Whether trials are happening in other countries?...Why does this need to be conducted among MSM in India…? (MSM, KI, India) |
| ***Early trial cessations***  They [PWID] hate them [medical providers]; they feel persecuted by them; they feel belittled and judged, and I don’t blame them because they are…. There has to be some repair done. I think they’ve widened the gap of mistrust. I might feel like I was lied to (PWID, KI, Canada)  The way I found out about this was through the media so initially there was some hostility on my part because I felt well, I’m in this study, if I’m finding out through the media that means that someone knew a week ago at least, you know what I mean? It’s very scary when you find out about something that you’re involved in, not from the people that you were working with but from an outside source (Gay man, KI, Canada)  In general, you know people get really psyched up and invested in a trial and they really want things to work. And for something to end early, either because there’s some indication of potential harm or because... the statistics show that it will never show effectiveness or they’re underpowered or whatever. But in all those situations people seem to be always caught by surprise, even though that’s something that as a researcher I know is always a possibility. I don’t think we communicate that, you know, that possibility as well as we need to (CSO rep., KI, South Africa)  They never actually said that anything like this could possibly happen, but of course if they did nobody would take the trial (Former trial participant, KI, Canada) |
| **Theme 3. Meaningful Community Stakeholder Engagement** |
| ***Early engagement***  An ideal model of community engagement would involve communities during protocol formulation stages to determine the community’s perceptions of the social value of the research (CSO rep., KI, South Africa)  My community wants to feel empowered; they want to feel engaged. Engage them in the actual setting up of the trial, in recruiting for the trial. Include them in every aspect (PWID, KI, Canada)  Communities need to be engaged more over the life of a trial…not only during community meetings when trials are recruiting and then again when results are going to be announced: this does not constitute meaningful community involvement (CSO rep., KI, South Africa)  And it seems like decisions are getting made in the Chief Council, but it’s some little old lady cooking soup in the back of the center who actually everyone checks in with first; You can’t really say, ‘I’m 30-years old; okay, Elders, come get educated by me’; I mean, that’s not respectful” (Aboriginal community, FG, Canada)  A mistake is if you keep your cards close to your chest and don’t really talk to people and don’t engage the community and don’t engage local stakeholders, and then something goes wrong and then you go knocking on people’s doors it’s a very different situation. It looks like you just want them when things get rough – so engage them throughout the whole process….talk to them and don’t delay community engagement and stakeholder engagement and communication (CSO rep., KI, South Africa) |
| ***The breadth of “community” representation***  The whole community, everyone should be involved; that way it’s being tested so that all populations can say this is something that is correct data (Female sex worker, KI, Canada)  Since I am working in a CBO, [trialists] invited me [for a consultation meeting]; others thus do not know about this and could not participate (Community rep., KI, India)  Advocates are seen as the necessary noise that should come after the agenda has been drawn, instead of being the ones drawing some of the agenda because they are so much in touch with communities (CBO rep., KI, South Africa)  One evolution of community engagement is that people don’t feel that that is enough; just having as group in place does not necessarily mean that you are doing appropriate community engagement (CSO rep., KI, South Africa)  Often negative perceptions about trials emerge because of how these are reported in the media, and indicate a failure on the part of the researchers to properly engage all relevant stakeholders (CSO rep., KI, South Africa) |
| ***Clear and appropriate roles***  My community wants to feel empowered; they want to feel engaged. Engage them in the actual setting up of the trial, in recruiting for the trial. Include them in every aspect (PWID, KI, Canada)  I think they must be consulted and they must be asked; but I’m a little bit skeptical of this, ‘let’s just involve communities for the sake of it.’ – They don’t have the skills; they’ve got other things to do, they’re busy; and I really do think there are other priorities for them other than to spend hours in consultation with prevention researchers.” (CSO rep., KI, South Africa)  If you have people at the table not because they are going to contribute in work but because they just want to be there, then you are going to expand the amount of work that needs to get done and the complexity of it, but not necessarily improve the outcome. (CSO rep., KI, South Africa)  People should have a say in decision making but you will have a futile trial that closes early if you turn over key decision-making to people who do not understand how science works and why trials are designed in particular ways (CSO rep., KI, South Africa)  So, when things get difficult and you need friends; or you have a hard time like when a trial closes early; you already have people who have a relationship with you, who are aware of your intentions; can be the voice in the community and with the media and with other stakeholders to help get the message out and do some damage control (CSO rep., KI, South Africa)  No proper information was given why they were no longer calling us… A meeting that was scheduled was cancelled. I could not face my community people when I had already spread messages about the trial coming. They were asking, ‘What is happening now? You asked us to come for meetings and now no noise’. I was very angry at that time. I felt as though we were being used – like a ‘use-and-throw’ [disposable object]” (MSM CBO, KI, India)  I also think the CAB’s being constituted by the research community and being sustained by the research community introduces some bias to what they do (CSO rep., KI, South Africa)  I’ve seen many CABs, not all CABs, being empowered enough to engage actively in the research (CSO rep., KI, South Africa) |
| ***Benefits of engagement***  I felt a bit like a pioneer…I was actually quite proud to be part of something that could have some far-reaching impact” (Former trial participant, KI, Canada)  Even though I was a participant, I felt like I was an important part of the whole team. They didn’t just treat me like a patient or a research study participant. I felt like I was an important part of the whole process. I wasn’t just a guinea pig (MSM, KI, Canada)  Now we are having a club in our trial for our participants who want to create some kind of HIV/AIDS campaign in the future (Clinical trialist, KI, Thailand)  So, when things get difficult and you need friends; or you have a hard time like when a trial closes early; you already have people who have a relationship with you, who are aware of your intentions; can be the voice in the community and with the media and with other stakeholders to help get the message out and do some damage control (CSO rep., KI, South Africa)  A number of trials that have ended early or have had results that have been confusing have forced a different relationship between the groups where they both need each other to move this forward (CSO rep., KI, South Africa)  When a trial closes early, you already have people who have a relationship with you to help get the message out (CSO rep., KI, South Africa) |

CAB, community advisory board

CBO, community-based organization

CSO, civil society organization

FG, focus group

KI, key informant

MSM, men who have sex with men

PWID, person who injects drugs

rep., representative

TG, transgender woman
